# Appetite measures as correlates of clinical response in mood disorders treated with ketamine: systematic review

**DOI:** 10.3389/fnut.2025.1616859

**Published:** 2025-08-21

**Authors:** Jakub Słupski, Agnieszka Mechlińska, Adam Włodarczyk, Aleksander Kwaśny, Joanna Szarmach, Anita Słupska, Wiesław Jerzy Cubała

**Affiliations:** Faculty of Medicine, Department of Psychiatry, Medical University of Gdańsk, Gdańsk, Poland

**Keywords:** depression, TRD, appetite, ketamine, mood disorders, antidepressants

## Abstract

Mood disorders, including major depressive disorder (MDD) and bipolar disorder (BP), significantly impact global health, with MDD affecting over 300 million people and BP affecting approximately 2% of the world’s population. Ketamine, originally an anesthetic, has emerged as a promising treatment for patients with treatment-resistant depression (TRD), due to its unique pharmacological properties, such as N-methyl-D-aspartate (NMDA) receptor antagonism and anti-inflammatory effects. The potential of ketamine in treating depression has sparked debate regarding its effects on appetite. This paper aims to conduct a systematic review focusing on the complex interplay between ketamine treatment and appetite. A total of 78 references were identified from electronic databases: PubMed, Web of Science, APA PsycINFO, and EBSCOhost, with 5 meeting the inclusion criteria for this review, encompassing 678 participants. Appetite was assessed using both clinician-rated and self-reported scales. Two studies reported significant improvement in reduced appetite following ketamine or esketamine treatment; one reported no significant change; one found a paradoxical worsening of reduced appetite; and one noted minimal effect on increased appetite and atypical symptoms. The data presented suggest that in patients with treatment-resistant mood disorders, ketamine may contribute to the improvement of depressive symptoms, including those related to appetite, or may exhibit neutral effects on food consumption desire. Appetite measurement may be a valuable indicator of the antidepressant effect, facilitating signal detection for substances beyond traditional monoaminergic antidepressants. Despite limited data, establishing a confirmed link between appetite and antidepressants could aid in treatment planning, particularly for patients with metabolic disorders or those at risk of malnutrition, potentially improving adherence and outcomes in treatment-resistant mood disorders.

**Systematic review registration:**

https://www.crd.york.ac.uk/PROSPERO/view/CRD42024510640, identifier CRD42024588790.

## 1 Introduction

Mood disorders, including major depressive disorder (MDD) and bipolar disorder (BP) pose a significant global health burden. The prevalence of MDD is staggering, affecting over 300 million people worldwide, equivalent to approximately 4.4% of the world’s population (1) while BP affects around 2% of the world’s population and encompass a spectrum between severe elevated and excitable mood states (mania) to the dysphoria, low energy, and despondency of depressive episodes (2). Despite advances in psychiatric treatments, a subset of patients’ experiences treatment-resistant depression (TRD). Treatment resistance is commonly defined as an inadequate response to at least two trials of treatment.

It encompasses two to five antidepressant treatment failures, changes between different classes of antidepressants, pharmacological augmentation strategies, and the addition of non-pharmacological interventions (3). These individuals do not respond adequately to conventional antidepressant therapies. TRD affects up to 30% of adults with MDD, presenting a formidable clinical challenge (4). The economic and social impacts of TRD are profound, as it leads to decreased productivity, increased healthcare utilization, and impaired quality of life (5). Neuroinflammation is increasingly recognized as a key pathophysiological component in MDD. Elevated levels of pro-inflammatory cytokines—IL-6, TNF-*α*, IL-1β—have been observed in depressed patients and are associated with sickness behavior (anhedonia, fatigue, anorexia), overlapping with depressive symptoms. Microglial activation leads to the release of these cytokines and disrupts monoaminergic and glutamatergic signaling. Chronic inflammation alters the kynurenine pathway, reducing serotonin and increasing neurotoxic metabolites like quinolinic acid inducing N-methyl-D-aspartate (NMDA) receptor activation (6).

Ketamine is a therapeutic option that remains innovative. Originally developed as an anesthetic, ketamine has emerged as a promising alternative for patients who have not responded to standard treatments (7). Ketamine, with its chiral structure of esketamine and arketamine, metabolizes into norketamine via cytochrome P450 enzymes. As an NMDA receptor antagonist, it modulates glutamatergic neurotransmission, enhancing synaptic plasticity and altering neurotransmission, which contribute to its antidepressant effects (8). Ketamine also promotes neuroplasticity by stimulating brain-derived neurotrophic factor (BDNF) release and synaptogenesis, potentially underlying its sustained effects on mood and cognition (9). Ketamine exhibits antidepressant effects in TRD, partly via modulation of neuroinflammation: reduces levels of IL-6, TNF-*α*, and IL-1β in both animal and human studies; inhibits TLR4-mediated NF-κB signaling and reduces microglial activation; modulates the kynurenine pathway, favoring neuroprotective kynurenic acid over quinolinic acid (10, 11). Its anti-inflammatory properties, through microglial inhibition and cytokine modulation, further enhance its therapeutic potential by reducing neuroinflammation and alleviating depressive symptoms (12).

Alterations in appetite represent a fundamental symptom of depression, plausibly linked to systemic low-grade inflammation. Inflammatory cytokines interfere with hypothalamic appetite control, particularly in the arcuate nucleus: IL-1β and TNF-*α* reduce neuropeptide Y (NPY) and agouti-related peptide (AgRP), leading to anorexia. They simultaneously increase pro-opiomelanocortin (POMC) and corticotropin-releasing hormone (CRH), which are anorexigenic (13). This association holds particular interest within the context of ketamine’s mechanism of action, especially considering its anti-inflammatory properties (14). It is worth mentioning that dietary pattern alterations are common in mood disorders, affecting caloric intake, meal composition, taste, and quality sensation. Thus, appetite changes, one of the nine criteria for diagnosing a major depressive episode (MDE), may serve as a surrogate marker for assessing antidepressant response. Animal studies and non-depression-related human investigations have reported appetite loss as a potential side effect of ketamine (15, 16). However, other researchers (17–19) have highlighted that ketamine may offer efficacious options for treating MDD with minimal impact on appetite and weight. Although it may be far from perfect, the feasibility of appetite assessment acquisition may be of interest to detect antidepressant effect.

Ketamine’s role in mood disorders encompasses addressing neural circuitry and managing appetite dysregulation, providing hope to individuals with depression. In this paper, we aim to conduct a systematic review focused on the intricate interplay between appetite, depression, and ketamine, examining its benefits in MDD outcomes through appetite control.

Although numerous systematic reviews and meta-analyses have been conducted on the efficacy, safety, and tolerability of ketamine in the treatment of depression—including racemic ketamine and esketamine administered via various routes—none have examined its impact on appetite. Recent comprehensive syntheses have focused on symptomatic improvement, treatment response, remission rates, dose–response relationships, and reduction of suicidal ideation (20–31). Yet, appetite-related outcomes remain unreported. This gap is notable given the central role of appetite disturbances in depressive syndromes and the known psychotropic profile of ketamine, which could plausibly affect appetite regulation. To our knowledge, no systematic review to date has addressed this specific domain.

## 2 Materials and methods

This systematic review followed the guidelines of the Preferred Reporting Items for Systematic Reviews and Meta-Analyses (PRISMA) statement. The Supplementary material contain the PRISMA checklist and the search results. The PROSPERO Registry (CRD42024510640) registered the protocol for this systematic review.

### 2.1 Information sources, search strategy and selection process

February 2024, we searched PubMed, Web of Science, APA PsycINFO and EBSCOhost electronic databases using the primary PubMed query as follows: “*Appetite” AND* (“*Mood disorders” OR* “*Depression” OR “TRD” or “Treatment-resistant depression” OR* “*MDD” OR* “*MDE” OR* “*bipolar disorder” OR* “*BP*”) *OR* “*Bipolar Depression” AND* (*“Ketamine” or “Esketamine” OR* “*Arketamine”*). The query had its structure adapted for each database according to specific requirements or syntax nuances (Supplementary file 1).

The inclusion criteria were:

1. Primary research articles.
2. Studies regarding patients with major depressive disorder or depression in bipolar disorder according to DSM or ICD diagnostic criteria.
3. Participants were exposed to ketamine or its enantiomers.
4. Pre- and post treatment appetite outcome was available.
5. Only adult patients (age ≥ 18 years old).

Overall PICOS (Population, Intervention, Comparison, Outcome, and Study Design) for this manuscript are:

Population (P): adults (≥18 years old) with treatment-resistant mood disorders (MDD and BP) diagnosed according to DSM or ICD criteria.

Intervention (I): treatment with ketamine (or its enantiomers) administered via intravenous or nasal spray.

Comparison (C): placebo or baseline condition; in some studies, treatment as usual (e.g., mood stabilizers).

Outcomes (O): changes in appetite measures [e.g., Montgomery-Asberg Depression Rating (MADRS) appetite item, Patient Health Questionnaire (PHQ-9) appetite item, or other scales capturing appetite or neurovegetative symptoms] as correlates of antidepressant response.

Study Design (S): randomized controlled trials, post-hoc analyses of randomized control trials (RCTs), and open-label single-arm studies.

### 2.2 Data collection process

The search process, the screening of abstracts and titles, and the reading of eligible full-text articles were done by three reviewers (J. S., A. K., A. M.). They resolved any disagreements with the help of the project co-supervisor (W. J. C.)

### 2.3 Study risk of bias assessment

To assess the risk of bias in non-randomized studies with interventions, we employed the Newcastle–Ottawa Scale (49). This scale assigns a maximum of nine points based on three criteria: selection (four stars), comparability (two stars), and outcomes (three stars). Studies scoring seven points or higher are considered “good quality.” For randomized trials, we evaluated the risk of bias using a revised tool to assess the risk of bias in randomized trials – RoB2 (32). This assessment considered factors such as sequence generation, allocation concealment, blinding, missing outcome data, selective reporting, and other potential biases. The risk of bias was categorized as “low,” “some concerns,” or “high.” We used the Robvis tool to visually present the results from randomized trials (33). Two independent reviewers evaluated the risk of bias for each study (J. Sz., J. S.) and any conflicting information was resolved with input from the project co-supervisor (W. J. C.).

Assessment of heterogeneity and publication bias was not performed, as this review was conducted as a qualitative systematic review. A meta-analysis was deemed inappropriate due to potential overlap in data sources across studies and the lack of a consistent, direct link between intervention and outcome that aligned with the reviews’ PICO framework. Instead, findings from the included studies—across diverse patient populations, study designs, and outcome measures—were qualitatively examined to explore how these factors might influence the reported outcomes. The strength of the overall evidence was evaluated by assessing its robustness and by identifying the specific populations and contexts represented within the included studies. We acknowledge that no formal assessment of publication bias or small study effects was performed in this review. Given the small number of included studies (*n* = 5) and the heterogeneity of designs and outcome measures, statistical methods such as funnel plots or Eggers’ test would have limited interpretability and risked producing misleading results.

### 2.4 Certainty of evidence

No formal framework (e.g., GRADE) was applied to assess certainty in the body of evidence. Instead, confidence in findings was evaluated narratively based on study quality, risk of bias, consistency of outcome direction, directness of evidence, and limitations in sample size and outcome heterogeneity.

## 3 Results

### 3.1 General characteristic of selected studies

**Figure 1:**
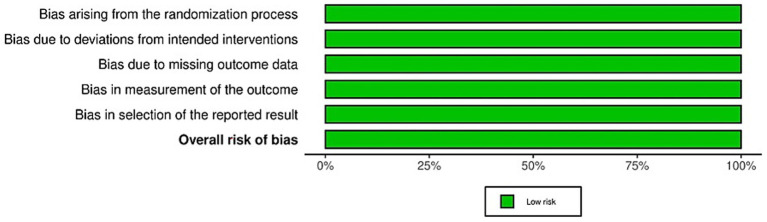
PRISMA 2020 flow diagram representing the search strategy and the process of including studies for analysis. From: Page et al. (48).

A total of 78 references were identified, with 66 undergoing review, leading to the exclusion of 58 papers that did not meet the inclusion criteria. The detailed screening process is illustrated in the PRISMA flow chart (Figure 1). Excluded articles were omitted for the following reasons: article did not focus on the topic of this review and investigated ketamine in the adolescent patient (1), research was based on data extracted from 4 other articles, of which 2 were included (1), no follow up with appetite measures (1).

Five studies were included in the review, comprising a total of 678 participants: 2 randomized, placebo-controlled studies (34, 35), 1 *post-hoc* analysis of data from 2 multicenter RCTs (36), 1 open-label, single-arm study (37), and 1 post-hoc exploratory analysis (38) including 2 of the RCTs mentioned in the first place - we decided to include this analysis as it presents separated approach to psychometric evaluation and includes population of MDD subjects. No studies for arketamine were identified. All studies included are presented in Table 1, which details study design, sample size, diagnostic criteria, appetite measures, and main findings.

**Table 1:** Included studies summary.

Author	Study design	Participants	Inclusion criteria	Intervention	Appetite outcome measures	Main findings
Diazgranados (34)	Randomized, placebo-controlled, double-blind, crossover, add-on study	*n* = 18 (12 female) BP I *n* = 8 BP II *n* = 10	Treatment-resistant depression (TRD) in the course of bipolar disorder (BP) I or II without psychotic features, Montgomery-Asberg Depression Rating Scale (MADRS) 20 or more at screening and baseline. Treatment resistance defined as failure to at least 1 adequate antidepressant trial AND failure to lithium or valproate	Lithium/valproate + single IV infusions of ketamine (0.5 mg/kg) vs. placebo	MADRS reduced appetite item (item 5)	Reduced appetite scores were significantly increased
Zarate (35)	Randomized, placebo-controlled, double-blind, crossover, add-on, single-center study	*n* = 15 (8 female) BP I *n* = 9 BP II *n* = 6	Treatment-resistant depression in the course of BP I or II without psychotic features, MADRS 20 or more at screening and baseline. Treatment resistance defined as failure to at least 1 adequate antidepressant trial AND failure to lithium or valproate	Lithium/valproate + single IV infusions of ketamine (0.5 mg/kg) vs. placebo	MADRS reduced appetite item (item 5)	Reduced appetite was not significantly improved on ketamine
Vande Voort (37)	Single-arm, open-label	*n* = 12 (11 female) MDD *n* = 9 BP I *n* = 1 BP II *n* = 2	TRD in MDD or BP I/II without psychotic features. Treatment resistance defined as failure to respond to at least two therapeutic trials of antidepressants or mood stabilizers (for patients with bipolar disorders)	6 Ketamine (0.5 mg/kg) IV infusions in 2 weeks acute phase, then 4 ketamine (0.5 mg/kg) IV infusions once weekly - remitters only	MADRS neurovegetative factor (items 3–5) PHQ-9 changed appetite item (item 5) - screening only	Significant changes in neurovegetative factor (including appetite, sleep and inner tension) were noticed after acute phase observation in remitters (*p* ≤ 0.001) and were not seen in non-remitters.
Park (38)	*Post hoc* exploratory analysis of data pooled from three separate, double-blind, placebo-controlled, crossover study	*n* = 68 (41 female)	TRD in MDD or BP I/II without psychotic features. Treatment resistance defined as failure to at least 1 previous antidepressant trial.	Single (0.5 mg/kg) IV infusion of ketamine vs. placebo; MDD patients were medication-free, BP patients remained on lithium or valproate	Hamilton Depression Rating Scale–Seasonal Affective Disorder (SIGH-SAD) and Scale for Atypical Symptoms (SAS) scoring	Ketamine demonstrated relatively smaller effects on sleep and appetite symptoms in comparison to typical and other atypical symptoms of depression
Borentain (36)	*Post hoc* analysis of data from two, phase 3 short-term, randomized double-blind active-controlled, multicenter studies	*n* = 565 (379 female)	TRD in MDD without psychotic features, IDS 34 or more at the baseline. Treatment resistance – failure to at least 2 antidepressants.	Esketamine (56 or 84 mg in flexible dosing) or placebo esketamine (56 or 84 mg in fixed dosing) or placebo	MADRS factor 2: anxiety and vegetative symptoms (inner tension, reduced sleep, reduced appetite, concentration difficulties)	MADRS Factor 1 and Factor 2 including appetite specifically improved over 4 weeks of treatment in comparison to Factor 3.

### 3.2 Risk of bias in the studies

**Figure 2:**
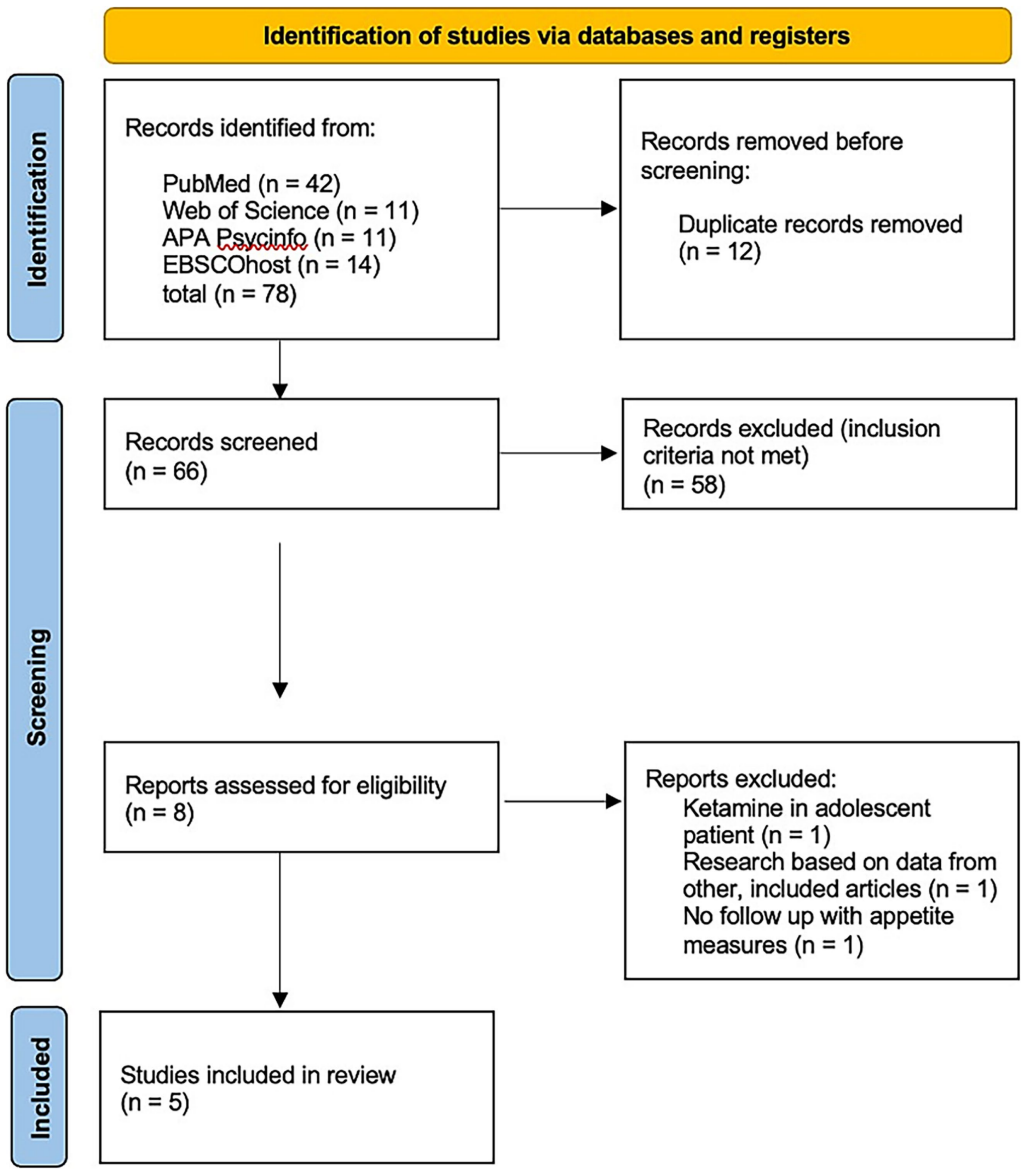
Traffic-light plot for risk of bias domains.

**Figure 3:**
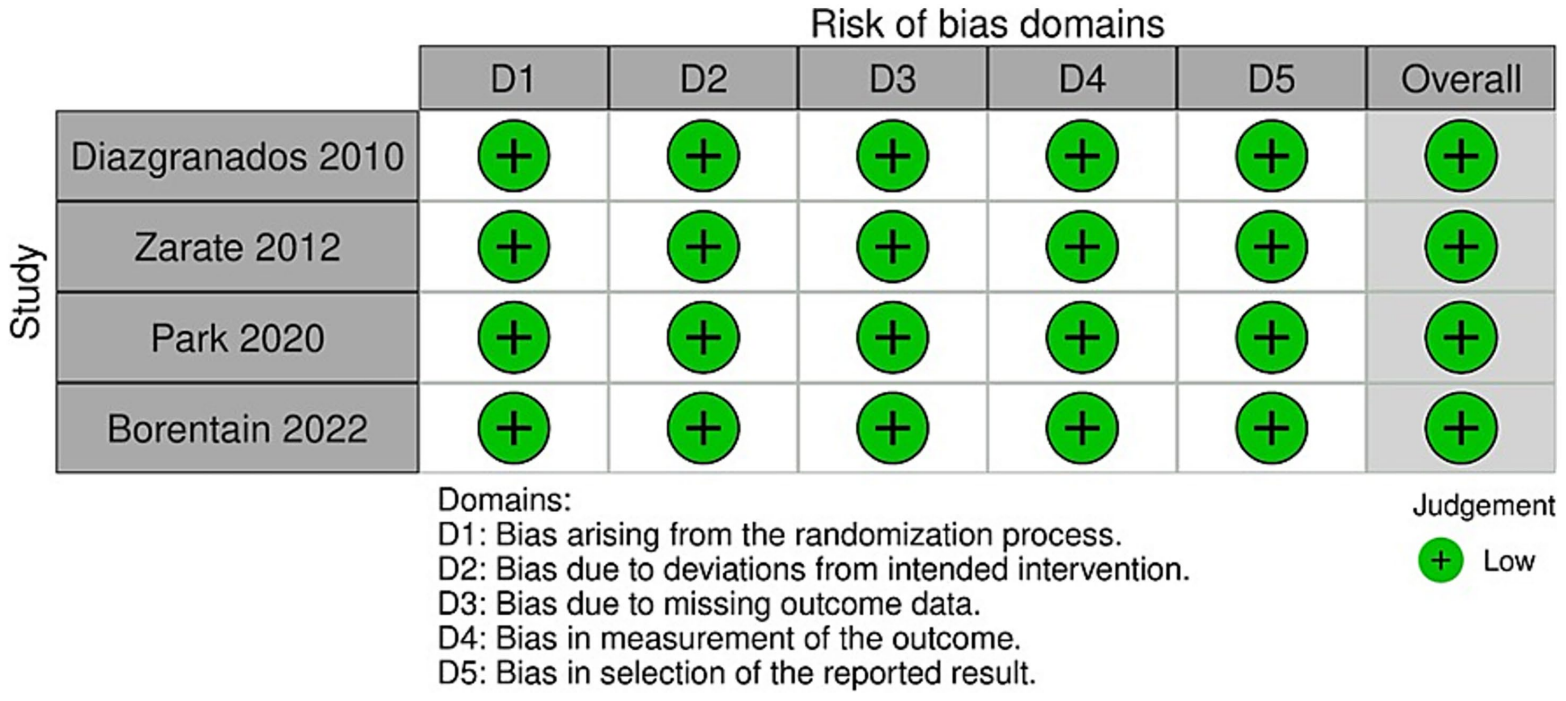
Summary plot for risk of bias domains.

Randomized trials were assessed according to the RoB 2 tool (a revised tool for assessing the Risk of Bias in randomized trials). Outcomes for RCTs are presented in Figures 2, 3. These show that most studies were rated as having either low risk or some concerns, mainly due to small sample sizes and limited blinding of outcome assessors.

Non-randomized trial (37) was assessed with NOS (16) receiving 3 of 4 stars for selection, 0 of 2 stars for comparability and 3 of 3 stars for outcome, resulting in 6 out of 9 stars in summary. This NOS score reflects a moderate quality for the study, with the major limitations as follows: the absence of a non-exposed cohort or control group, small sample size and limited diversity reducing generalizability. Also lack of blinding adds a significant risk of bias in outcome measures.

### 3.3 Study characteristics

1. Diazgranados et al. (34) conducted a single-center, randomized, placebo-controlled, double-blind, crossover, add-on study (Oct 2006 - Jun 2009) to determine whether an NMDA receptor antagonist shows rapid antidepressant effect in BP depression. Eighteen subjects were randomized and received either a subanesthetic dose of ketamine (0.5 mg/kg) or 0.9% of saline. MADRS score was the primary outcome measure. Regarding appetite MADRS item 5 was evaluated and an analysis of individual MADRS items showed that reduced appetite scores were significantly increased while all other symptoms decreased.
2. Zarate et al. (35) performed a replication of previously described study involving 15 subjects with BP. In this study no increase of reduced appetite score was noted, however reduced appetite and decreased sleep were the only depressive symptoms domains without improvement.
3. In an open-label study by Vande Voort et al. (37) twelve TRD inpatients were treated with intravenous (i.v.) ketamine infusions during acute-phase followed by four weeks of continuation treatment. The severity of the symptoms was measured by a clinician with MADRS, combining changes in appetite, sleep, and inner tension into one factor. All patients reported an overall improvement after the first infusion or third infusion, with five patients achieving remission and seven patients responding to treatment. Both groups did not differ in the neurovegetative factor at baseline, however robust and statistically significant changes were noticed after acute phase observation in remitters (*p* ≤ 0.001) in contrast to non-remission group.
4. Park et al. (38) conducted a post-hoc exploratory analysis of data pooled from three separate, double-blind, placebo-controlled, crossover studies. 68 subjects with TRD in MDD or BP were included into the analysis. BP patients remained on mood stabilizing treatment while MDD participants followed a wash-out period and both groups received single subanesthetic (0.5 mg/kg) infusion of ketamine hydrochloride. Psychometry was evaluated using MADRS and Hamilton Depression Rating Scale – Seasonal Affective Disorder (SIGH-SAD) as well as Scale for Atypical Symptoms (SAS) questionnaires which enabled the assessment of atypical symptoms including increased appetite and eating, weight gain and carbohydrate craving. In the end ketamine manifested relatively smaller effects on sleep and appetite symptoms in comparison to typical and other atypical symptoms of depression.
5. Borentain et al. (36) study based on two short-term, double-blind, randomized, active-controlled, multicenter studies of esketamine nasal spray including 565 patients with treatment-resistant MDD. The aim of this study was to examine and validate the dimensions of the MADRS in individuals with TRD and assess the changes in the baseline factors over a 4-week period of esketamine therapy. Three MADRS factors were distinguished: Factor 1 - affective and anhedonic symptoms (apparent sadness, reported sadness, lassitude, inability to feel), Factor 2 - anxiety and vegetative symptoms (inner tension, reduced sleep, reduced appetite, concentration difficulties), Factor 3 - hopelessness (pessimistic thoughts, suicidal thoughts). In result MADRS Factor 1 and Factor 2 including appetite specifically improved over 4 weeks of treatment in comparison to Factor 3.

### 3.4 Appetite measures

The appetite measures were conducted in five studies, MADRS scale was employed consistently across four of these studies to assess this factor (34–38). Data from the publications are collectively presented in Table 1. In 1 out of 5 studies, a statistically significant worsening of appetite was observed following intravenous administration of ketamine in patients with bipolar disorder compared to placebo. Significant improvement was noted in other depressive symptoms (34). Appetite improvement was observed in two studies (36, 37). Comparing participants who achieved remission during acute phase treatment (*n* = 5) to non-remitters after intravenous ketamine treatment (*n* = 7), a statistically significant overall improvement was observed in MADRS scores (−79.1 ± 13.0 vs. − 14.6 ± 11.0; *p* < 0.001) and in neurovegetative symptoms (−84.3 ± 20.4 vs. − 8.4 ± 54.4; *p* < 0.001) (37). After 4-weeks, changes in appetite were significant with the treatment. Factor 2 scores improved at all post-baseline time points, with esketamine plus antidepressant proving more effective than antidepressant plus placebo (*p* < 0.05), significantly affecting symptoms such as reduced appetite (36). In two studies, significant improvement in appetite was not observed (35, 38).

In the included studies, appetite was variably assessed using both observer-rated and self-report measures.

1. Observer-rated instruments:

The MADRS includes item 5 (reduced appetite). This is rated by clinicians and reflects decreased appetite. Factor analysis of MADRS also includes appetite within the “neurovegetative” domain.

2. Self-report instruments:

The PHQ-9 includes item 5, which assesses changes in appetite, capturing both increased and decreased appetite, though in Vande Voort et al. (37) it was only used at screening. The SIGH-SAD and SAS (38) include items on increased appetite, carbohydrate craving, and weight gain, allowing evaluation of hyperphagic symptoms.

Based on these instruments, we categorized appetite-related outcomes as:

1. By source:

*Observer-rated*: MADRS item 5, MADRS-derived neurovegetative factors

*Self-report*: PHQ-9, SAS, SIGH-SAD

2. By direction:

*Reduced appetite*: MADRS item 5, PHQ-9 decrease, SIGH-SAD

*Increased appetite*: SAS (e.g., carb craving), PHQ-9 increase

#### 3.4.1 Of the observer-rated studies

Diazgranados et al. (19) showed a paradoxical worsening of reduced appetite scores (i.e., appetite remained poor or worsened). Zarate et al. (35) found no significant improvement in reduced appetite. Vande Voort et al. (37) demonstrated improvement in a composite neurovegetative factor, which included appetite. Borentain et al. (36) found that MADRS Factor 2 (reduced appetite among other symptoms) improved significantly over 4 weeks.

#### 3.4.2 Regarding self-report data

PHQ-9 (37), although limited to screening, provided directional context for appetite changes but not post-treatment comparison. SIGH-SAD and SAS (38) allowed the assessment of increased appetite and cravings, indicating that ketamine had smaller effects on atypical neurovegetative symptoms compared to core depressive features.

## 4 Discussion

The data presented in our systematic review suggest that in patients with treatment-resistant mood disorders, ketamine may contribute to the improvement of depressive symptoms, including appetite, or show neutral effects on the desire for food consumption. The challenge in observing significant changes in appetite scores highlights the variability in symptom response across different patient groups and treatment protocols. In the study by Diazgranados et al. (34), appetite was the only symptom that did not show significant improvement after intravenous ketamine administration; on the contrary, its decrease was observed. However, considering the overall number of study participants who responded positively to ketamine treatment, improvement in appetite appears achievable, especially when combined with another antidepressant medication (36). Appetite changes, as measured by both self-report and observer-rated tools, showed heterogeneous results across studies. Notably, observer-rated tools predominantly capture reduced appetite, whereas self-report measures, such as SAS and SIGH-SAD, offer insight into increased appetite and atypical features. This distinction is essential, as appetitive changes may be linked to distinct neurobiological pathways. Ketamine’s effects on mood symptoms may not extend uniformly to appetite, particularly when hypophagic versus hyperphagic symptoms are governed by divergent mechanisms. The inconsistency in outcome could reflect methodological limitations, but also true clinical variability, including divergent appetitive symptoms across depressive subtypes (melancholic vs. atypical). Future studies should incorporate dual-assessment strategies (clinician- and self-rated) to capture the full spectrum of appetite-related effects of ketamine.

The potential of ketamine in treating depression, although promising due to its anti-inflammatory properties, initiates a debate regarding its effect on appetite. Mood disorders frequently entail alterations in appetite, and treatment with antidepressants can assist in managing these fluctuations. Pharmacological differences between antidepressants can lead to varying susceptibility to weight gain and metabolic disturbances. Some antidepressants may increase appetite and impair satiety, raising the risk of overweight or obesity, while others may have anorexigenic effects. Depending on the patient’s health condition, fluctuations in appetite and resulting weight changes can affect the patient’s adherence to the prescribed pharmacological regimen (17). Moreover, depressive symptoms like sorrow and a sense of despair can influence digestive processes, intensifying both weight and energy decline. Furthermore, undernourishment can impede the recuperation process, functioning, and overall quality of life among individuals with MDD. This association between appetite alterations, undernourishment and MDD can result in persistent concurrent conditions, with each condition exacerbating the severity of the other (39). In a study comparing nutrient intake in individuals with TRD before and after ketamine treatment, it was observed that participants’ diets were significantly nutrient-poor, and nutrient intake decreased even further post-treatment. This may indicate fluctuations in overall food intake or changes in appetite for specific nutrients. For instance, carbohydrate consumption decreased following ketamine treatment (19). In a cross-sectional study, individuals with mood disorders who attempted suicide showed lower serum triglycerides and reduced adiposity (lower BMI and waist circumference) compared to those without a history of suicide attempt. The study did not explicitly address malnourishment among those who attempted suicide. Additionally, being cross-sectional, it could not determine whether decreased triglycerides preceded mood episodes (40).

The neurobiological mechanisms that may explain appetite-related effects of ketamine are still under investigation. The multifaceted relationship between appetite and the brain involves various neural circuits, hormones, and neurotransmitters, while the precise way in which ketamine may regulate appetite remains unclear. Studies indicate that depending on the type of NMDA receptor modulation, food consumption can be inhibited by agonists or stimulated by antagonists of the receptor. Ketamine, as an NMDA antagonist, might influence feeding behavior through hypothalamic pathways or dopamine-mediated reward systems, but the net effect appears to depend on individual biology and symptom profile (41). Glucose uptake in the small intestine is vital for appetite control, functioning through diverse pathways. The glucostatic theory posits that glucose acts as an immediate satiety cue by influencing plasma glucose concentrations. Additionally, it contributes to the body’s homeostatic mechanisms, offering input to the brain to manage food consumption and sustain blood glucose levels. Nevertheless, hedonic regulation can supersede these processes, as pleasurable glucose ingestion stimulates dopamine release in the brain, impacting appetite (42). Diverse brain regions are involved in glucose sensing and regulation, including the hypothalamus, brainstem, cerebral cortex, nucleus accumbens, prefrontal cortex, and amygdala (43). Shank3, a protein located in the post-synaptic density, is linked to bipolar disorder’s pathophysiology and has been investigated in the context of ketamine’s antidepressant effects on individuals with bipolar depression. Higher levels of Shank3 before ketamine treatment are associated with better responses to ketamine, as well as correlate with increased glucose metabolism in the hippocampus and amygdala following ketamine treatment (44). Such findings suggest that ketamine’s appetite-related effects could be secondary to its modulation of metabolic and reward-related signaling in specific brain regions, particularly in patients with abnormal baseline metabolism. The hypothalamus coordinates homeostatic regulation by integrating signals from peripheral organs such as the gut and adipose tissue, with hormones like leptin and ghrelin signaling hunger and satiety. Simultaneously, the mesolimbic dopamine system, encompassing regions like the ventral tegmental area (VTA) and nucleus accumbens, is crucial in processing food reward and motivating eating behavior, mediated by dopamine. Stress, mood, and emotions influence appetite, with ghrelin implicated in stress-induced food intake, while impulsivity and cognitive factors affect food reward behaviors through dopaminergic activity in the brain’s reward circuitry (42). Furthermore, the insulin signaling pathways within the brain play a role in regulating food intake and energy balance, thereby influencing appetite regulation. Aberrant insulin signaling has been associated with mood disorders like depression, as evidenced by animal models exhibiting behaviors resembling depression, which can be ameliorated through insulin therapy. These overlapping systems may help explain why ketamine, despite its rapid antidepressant effects, has an inconsistent or delayed impact on appetite symptoms – especially if these symptoms are linked to metabolic rather than affective dysregulation (45). Comprehending the complex interactions among brain regions, glucose metabolism, and appetite regulation is essential for formulating efficacious strategies to manage appetite and tackle concerns such as overeating, obesity, and mental disorders.

The association between depressive symptoms, heightened appetite, body mass index (BMI), and insulin resistance has been underscored (46). Niciu et al. (47) demonstrated that higher BMI may serve as a significant indicator of improvement after ketamine treatment, particularly in the acute phase, and patients with higher BMI may not sustain the initial response to antidepressant medication. It can be inferred that considering BMI may aid in selecting a clinically effective dose of ketamine.

Appetite changes may serve as a useful measure for assessing the antidepressant effect, particularly for substances that differ from traditional monoaminergic antidepressants. The development of rapid-acting antidepressants (RAADs) highlights the psychometric limitations of traditional outcome measures such as the MADRS and the Hamilton Depression Rating Scale. Novel substances in development often exhibit distinct antidepressant effects, including antianhedonic and antisuicidal properties. Therefore, appetite measurement may help refine and correct the observed response in these ‘gold-standard’ measures.

Several constraints of this systematic review require consideration. The key issue is the limited number of scientific studies regarding the impact of ketamine on appetite, complicating the drawing of definitive conclusions about potential clinical implications. Moreover, the small number of available studies correlates with a limited number of participants, restricting the ability to accurately assess the desired effects. Gray literature sources such as trial registries (e.g., ClinicalTrials.gov), preprint servers (e.g., medRxiv), dissertations, or conference proceedings were not systematically searched.

Good practice in depression research and development involves setting measures based on a rater-based approach complemented by patient-reported outcomes. RAADs may require a variety of rater-based outcomes, as some measures may be biased and miss the signal. Thus, identifying feasible measures is crucial at all stages of the development process. Considering the increasing number of individuals suffering from mood disorders, including those with treatment-resistant conditions and associated metabolic disorders, it is crucial to focus future research on potential correlations between rapid-acting antidepressants and appetite. Clear information about such correlations could help clinicians propose appropriate treatments for depressive disorders while simultaneously reducing the risk of metabolic complications.

## 5 Conclusion

The data from our systematic review suggest that ketamine may contribute to the improvement of depressive symptoms, including appetite, in patients with TRD. However, studies in this field are lacking, what creates an opportunity for further exploration of the extent to which appetite can serve as a measure of positive antidepressant response to treatment. A verified correlation between appetite and antidepressants may assist in treatment planning, particularly for patients with metabolic disorders or those at risk of malnutrition. This could enhance treatment adherence and improve the likelihood of positive outcomes in patients with treatment-resistant mood disorders. In addition to monitoring appetite as a clinical signal, targeted interventions may help manage nutrition-related risks during ketamine treatment. Patients exhibiting reduced appetite may benefit from early dietary assessment and structured nutritional support, particularly those with low BMI or poor baseline dietary intake. For individuals with increased appetite or carbohydrate cravings, behavioral counseling and dietitian-guided regulation of glycemic load may reduce the risk of post-treatment weight gain and insulin resistance. Moreover, integrating nutritional psychoeducation and appetite monitoring into treatment planning could enhance engagement, especially in individuals with atypical features or comorbid metabolic disorders. These approaches may help clinicians anticipate and manage appetite-related side effects, thereby improving both psychiatric and physical outcomes.

## Data Availability

The original contributions presented in the study are included in the article/Supplementary material, further inquiries can be directed to the corresponding author.
