# Correction: Appetite measures as correlates of clinical response in mood disorders treated with ketamine: systematic review

**DOI:** 10.3389/fnut.2025.1699569

**Published:** 2025-09-24

**Authors:** Jakub Słupski, Agnieszka Mechlińska, Adam Włodarczyk, Aleksander Kwaśny, Joanna Szarmach, Anita Słupska, Wiesław Jerzy Cubała

**Affiliations:** Faculty of Medicine, Department of Psychiatry, Medical University of Gdańsk, Gdańsk, Poland

**Keywords:** depression, TRD, appetite, ketamine, mood disorders, antidepressants

In the published article, there was an error in the **Acknowledgments** statement. The institution was incorrectly written as, “Polish Agency for Medical Research”. The correct institution should have been written as, “Medical Research Agency”. The corrected Acknowledgments statement appears below:

“Manuscript preparation was supported during The Harvard Medical School's Polish Clinical Scholars Research Training Program. Program participation was facilitated by Medical Research Agency.”

The original article has been updated.

