# Supplementary material for: Appetite measures as correlates of clinical response in mood disorders treated with ketamine: systematic review
Source: Front Nutr. 2025 Aug 21;12:1616859. doi: 10.3389/fnut.2025.1616859 (PMC12415402; doi:10.3389/fnut.2025.1616859)
Supplement: Supplementary file 1 [file Table_1.DOCX]

PubMed

„Appetite” AND („Mood disorders” OR „Depression" OR "TRD" or "Treatment-resistant depression" OR „MDD” OR „MDE” OR „Bipolar disorder” OR „BP”) OR „Bipolar Depression” AND ("Ketamine" or "Esketamine" OR „Arketamine”)

Web of Science

TS=("Appetite") AND TS=("Mood disorders" OR "Depression" OR "TRD" OR "Treatment-resistant depression" OR "MDD" OR "MDE" OR "Bipolar disorder" OR "BP" OR "Bipolar Depression") AND TS=("Ketamine" OR "Esketamine" OR "Arketamine")

APA Psych Info
(Appetite) AND ("Mood disorders" OR "Depression" OR "TRD" OR "Treatment-resistant depression" OR "MDD" OR "MDE" OR "Bipolar disorder" OR "BP" OR "Bipolar Depression") AND ("Ketamine" OR "Esketamine" OR "Arketamine”)

EBSCOhost
„Appetite” AND („Mood disorders” or “depression” or “TRD” or “treatment-resistant depression” or “MDD” or “MDE” or “bipolar disorder” or “BP” or “bipolar depression” AND ("Ketamine" or "Esketamine" OR „Arketamine”)
